# Supplementary material for: Exploring the gut DNA virome in fecal immunochemical test stool samples reveals associations with lifestyle in a large population-based study
Source: Nat Commun. 2024 Feb 29;15:1791. doi: 10.1038/s41467-024-46033-0 (PMC10904388; doi:10.1038/s41467-024-46033-0)
Supplement: Supplementary file 1 — Supplementary information [file 41467_2024_46033_MOESM1_ESM.pdf]

Supplementary information for

Exploring the gut DNA virome in fecal immunochemical test stool samples reveals associations with lifestyle in a large population-based study

Paula Istvan*, Einar Birkeland*, Ekaterina Avershina, Ane S Kværner, Vahid Bemanian, Barbara Pardini, Sonia Tarallo, Willem de Vos, Torbjørn Rognes, Paula Berstad, Trine B Rounge#

#Corresponding author: Trine B Rounge, trinro@uio.no

Supplementary Figures

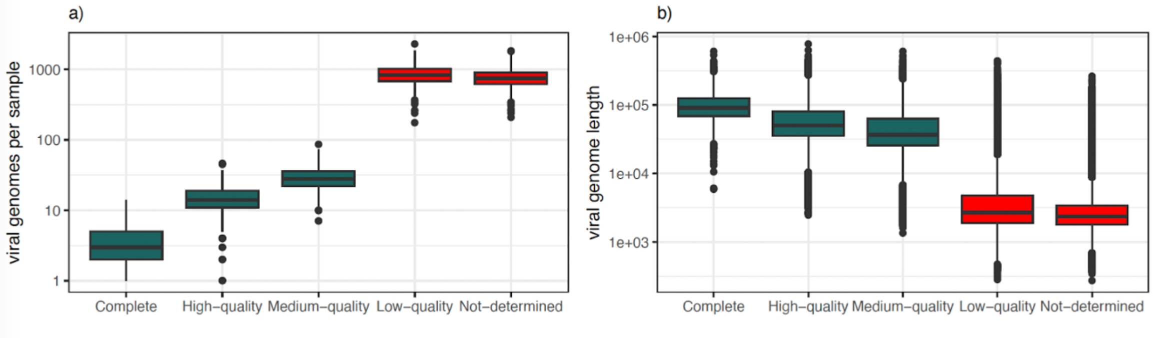

Supplementary fig. 1 | Viral genome quality a) Number of viral genomes identified per sample according to quality as determined by CheckV. b) Viral genome length according to CheckV-assigned quality category. The borders of the boxes denominate first (Q1) and third (Q3) quartiles, and the middle line represents the median. The whiskers extend to the most extreme point in the dataset but not further than Q1-1.5IQR (lower limit) and Q3+1.5IQR (higher limit). Data exceeding this value are shown as points. Green color represents viral genomes with medium or higher quality (corresponding to >50% completeness) were extracted and considered for further analysis, red ones were the viral genomes not considered.

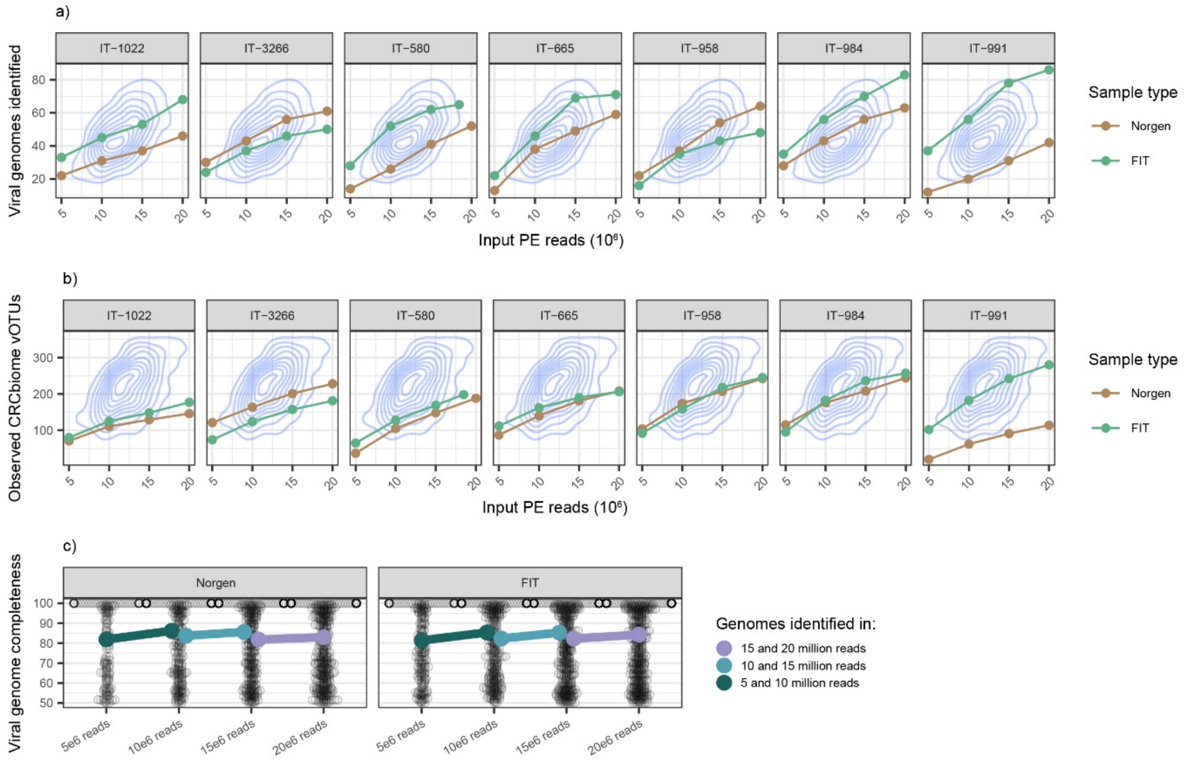

Supplementary fig. 2 | Pairwise comparison of stool samples collected using FIT and Norgen sampling kits. a-b) Panels show (a) the number of viral genomes of medium or higher quality identified, (b) number of CRCbiome vOTUs detected by read mapping in one set of paired FIT and Norgen samples. Each sample was subjected to random subsampling to 5, 10, 15, and 20 million reads (linked by lines), with the color indicating the type of sampling used. Density lines indicate the corresponding numbers for FIT samples in the CRCbiome study. c) Genomes identified in each sample at any level of subsampling were dereplicated to identify which genomes were identified across samples and across dereplication levels. Circles show the estimated completeness of

identified genomes at each level of subsampling. Colored lines indicate the shift in mean genome completeness for genomes identified in the same sample at both connected subsampling levels.

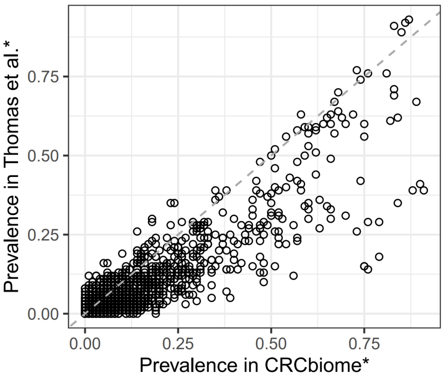

Supplementary fig. 3 | Prevalence of CRCbiome vOTUs in Thomas *et al.* Prevalence of CRCbiome vOTUs for samples drawn from CRCbiome and Thomas *et al.* matched by sequencing depth (n = 100 from each cohort). The dashed line indicates equal prevalence in the two populations. Asterisks indicate that the prevalence of each cohort is based on a subset of samples.

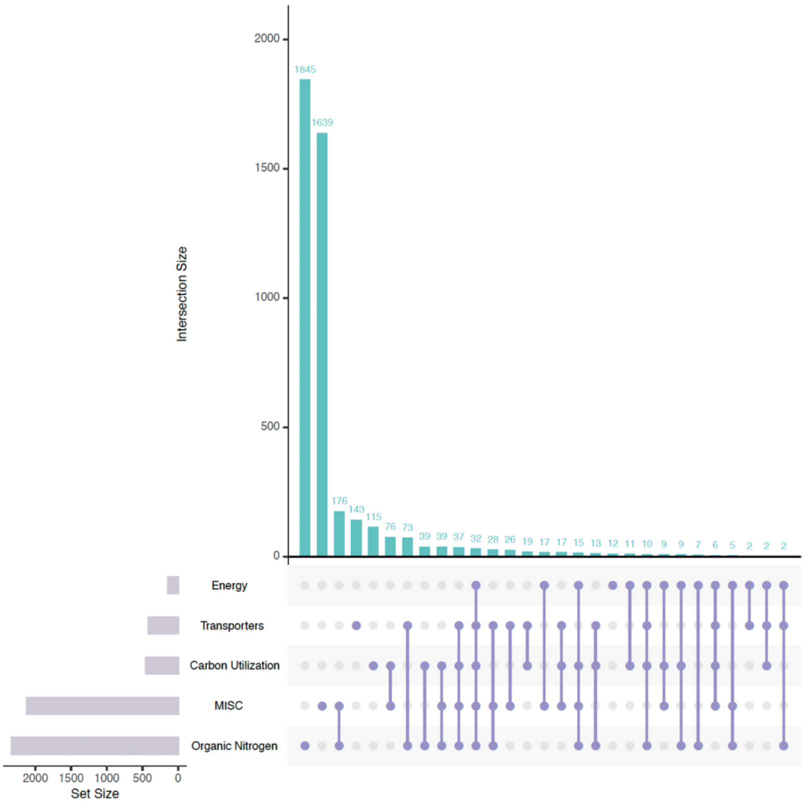

Supplementary fig. 4 | Frequency of AMG detection and intersections in vOTUs according to functional group. Filled dots with interconnecting vertical lines (purple) represent the intersections, and unfilled light gray dots represent sets that do not belong to the intersections. Vertical bars (light green) represent the numbers of vOTUs with AMGs for each intersection. Horizontal bars to the left (gray) depict the total number of vOTUs with AMGs in each functional group.

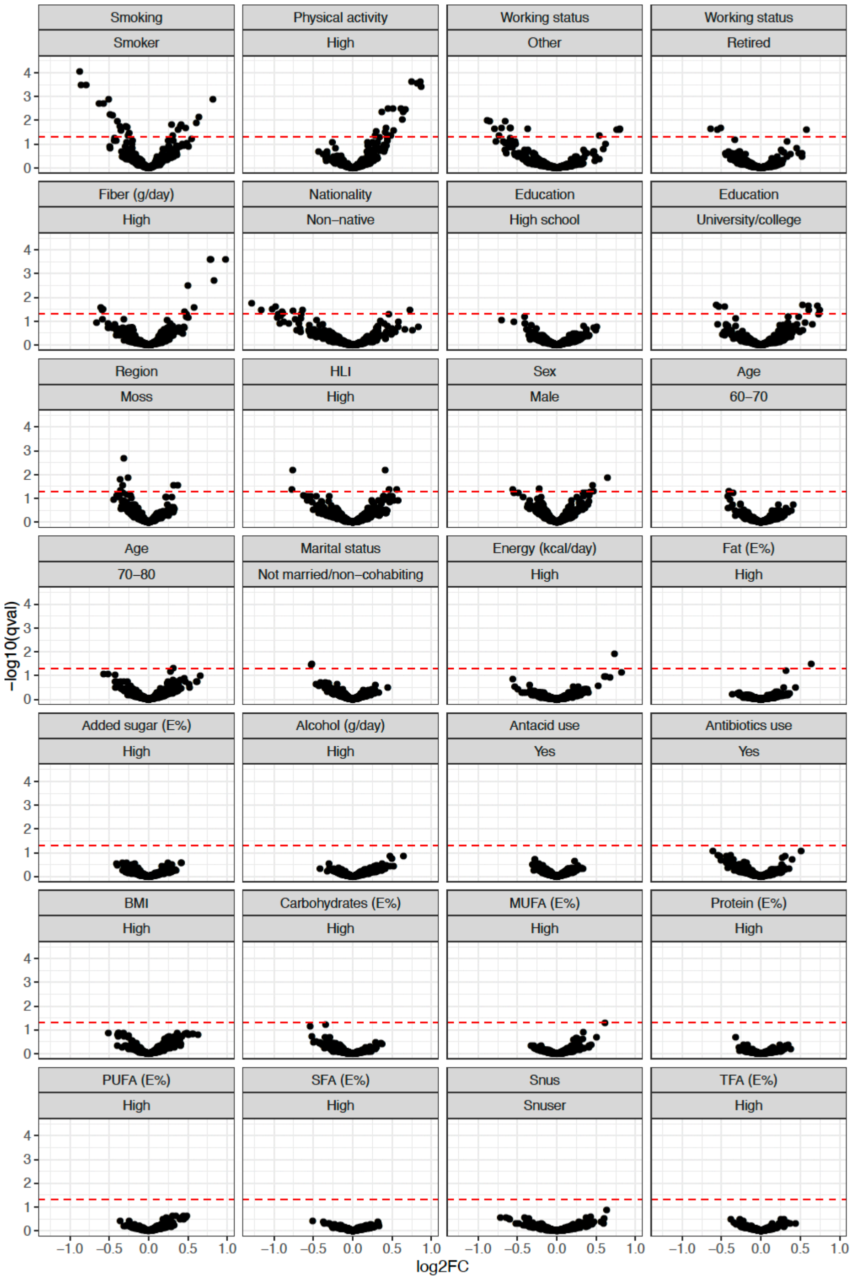

Supplementary fig. 5 | Volcano plots showing the relationship between effect size (log2 fold change) and significance level (q-value) for vOTUs for diet, lifestyle and demographic variables.

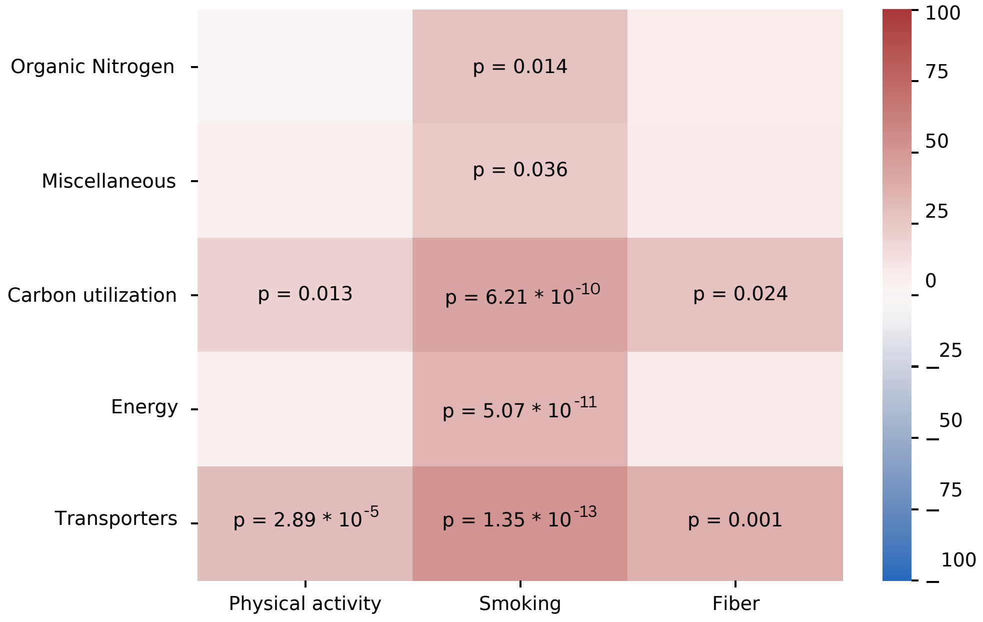

Supplementary fig. 6 | Difference in prevalence of AMGs in differentially abundant vOTUs compared to their prevalence in all vOTUs. The difference ranges from -100 (blue, absent in differentially abundant vOTUs but present all others) to 100 (red, present in all differentially abundant vOTUs and no others). Significance of differences in prevalence were assessed using a binomial test. FDR-corrected p-values are reported in the plot.

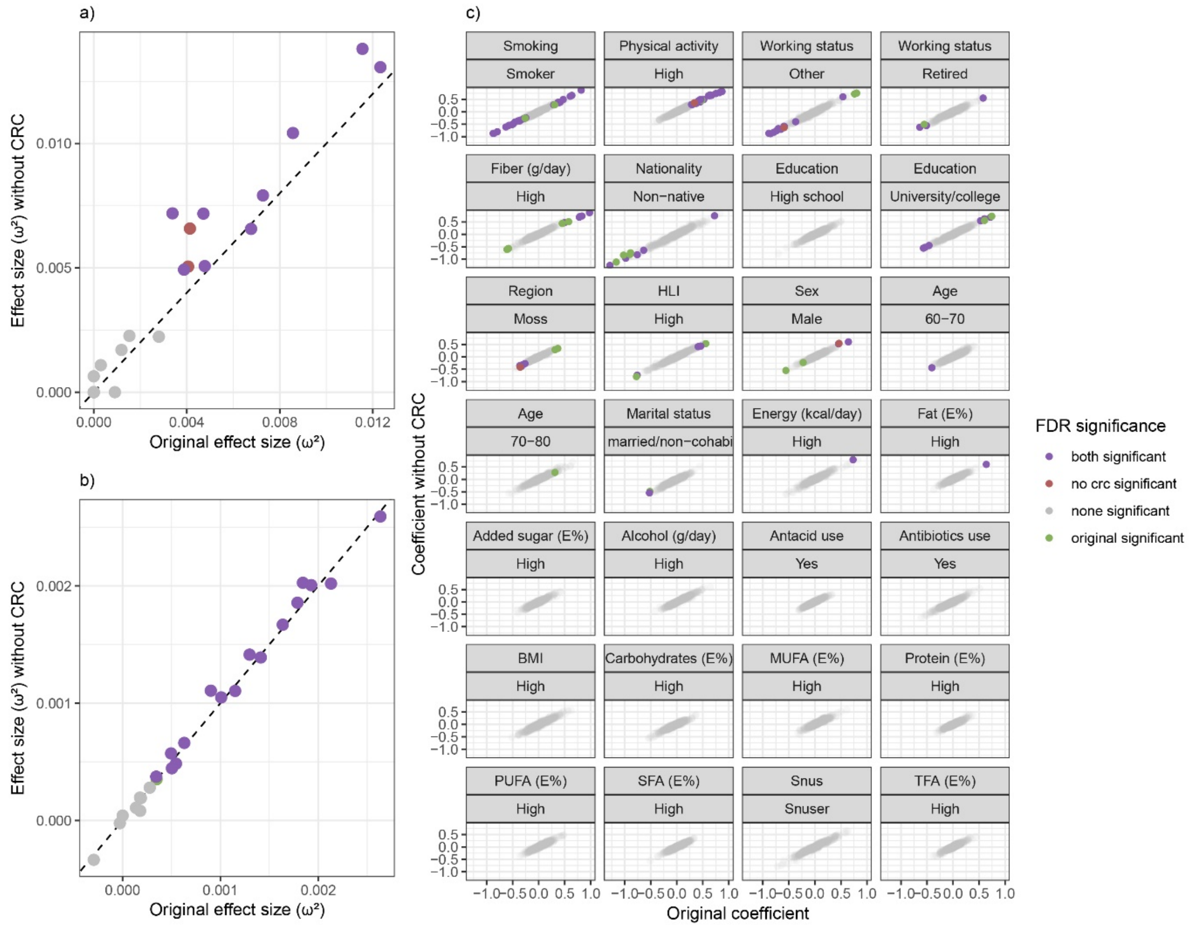

Supplementary fig. 7 | Sensitivity analyses excluding any participants with colorectal cancer. a-c) Effect sizes of associations between virome components and participant characteristics are presented for all participants (original effect size; x-axes) and for those without cancer diagnoses (effect size when excluding CRC; y-axes). Each point represents the effect sizes for associations for a) viral alpha diversity (inverse Simpson), b) beta diversity measured using Bray-Curtis dissimilarity, and c) individual vOTUs in relation to a particular lifestyle or demographic factor (in c) each lifestyle/demographic factor and the levels assessed are presented in a separate panel). Associations are color-coded according to statistical significance (FDR < 0.05) in the original analysis and when excluding CRC cases.

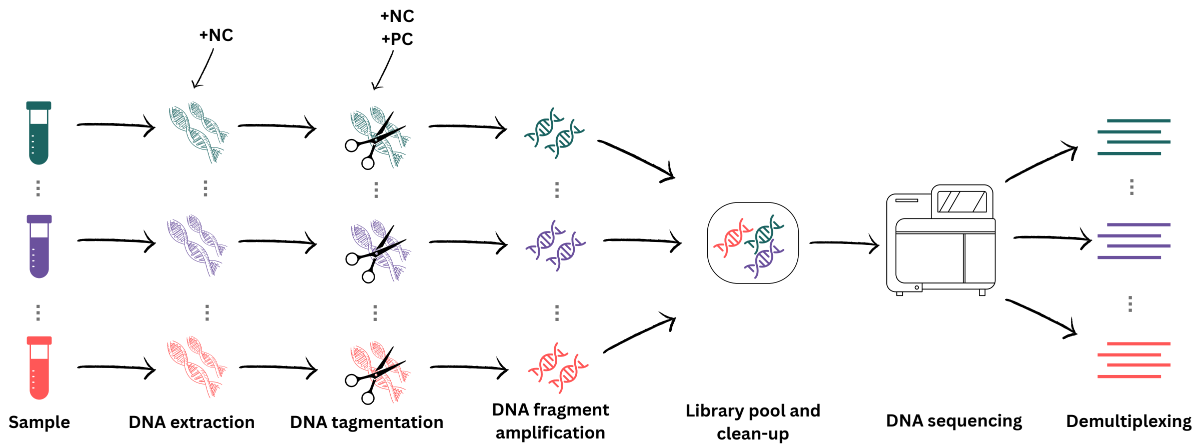

Supplementary fig. 8 | Schematic representation of wet lab sample processing for metagenomic sequencing data generation. Each sample DNA was extracted, tagmented (Nextera) and amplified using a combination of i5/i7 indexed Illumina primers before pooling into sequencing libraries, each comprising 240 samples. Libraries were then sequenced, and sequencing reads were demultiplexed. These reads were then filtered and used for the data analysis. ZYMOBiomics Microbial community DNA positive controls (PC, n = 2) were added prior to DNA tagmentation step, whereas negative controls (NC) were added both prior to DNA extraction (n = 6) and to DNA tagmentation step. The figure was created using *Canva*.

Supplementary Tables

Supplementary Table 1: Diversity indices

Index	Mean	SD	Median	Min	Max
Observed	222.54	69.32	221	41	505
Shannon	4.88	0.43	4.96	2.34	7.77
Inverse Simpson	93.54	43.71	89.15	2.79	245
Bray-Curtis dissimilarity	0.838	0.065	0.83	0.42	1

Supplementary Table 2: Eukaryotic viruses based on the references deposited in the Virus-Host database

CRCbiome-vOTU	Annotation Virus-Host database	Annotation INPHARED	Host	Prevalence (number of individuals)	Notes
vOTU05832	HPV-6	n.a.	Human	1	Reads mapping to HPV were detected in 59 individuals (1-10 reads: 51 individual; 11-50 reads: 7 individuals; > 500 reads: 1 individual)
vOTU07469	*Flyfo siphovirus* Tbat1_6	*Flyfo siphovirus* Tbat1_6	Pacific Flying fox	1	wrong host annotation in the Virus-Host database; it's a *Caudoviricetes* bacteriophage isolated from the Pacific Flying Fox https://journals.asm.org/doi/10.1128/mra.00038-22
vOTU09583	Human feces pecovirus	n.a.	Human	1	
vOTU10740	*Fadolivirus algeromassiliense*	n.a.	*Vermamoeba veriformis*	57	
vOTU14080	Fur seal faeces associated circular DNA virus	n.a.	fur seal	2	
vOTU14233	*Fadolivirus algeromassiliense*	*Acinetobacter phage* MD-2021a	*Vermamoeba veriformis*	532	indirect connections to both references, weaker connection to *Fadolivirus* (N221) than to *Acinetobacter* phage (N8)

Supplementary Table 3: Genome diversity by viral family

Family	vOTUs	genomes	Chao1 index	vOTUs/Chao1 index
Microviridae	528	726	5340	0.099
Suoliviridae	161	331	349	0.461
Intestiviridae	113	445	196	0.577
Steigviridae	109	280	229	0.476
Peduoviridae	97	132	243	0.399
Crevaviridae	33	382	55	0.600
Inoviridae	32	54	438	0.073
Winoviridae	26	214	35	0.743

Supplementary Table 4: Genome integration by viral genome family*

Family	Unintegrated	Integrated	Percent integrated	OR	95% CI	p value	FDR
Microviridae	177	549	24.4%	0.548	(0.46-0.651)	5.6E-13	6.14E-12
Suoliviridae	3	328	0.9%	0.016	(0.003-0.046)	9.5E-61	1.04E-59
Intestiviridae	1	444	0.2%	0.004	(0-0.021)	2.2E-87	2.39E-86
Steigviridae	5	275	1.8%	0.031	(0.01-0.073)	1.4E-47	1.55E-46
Peduoviridae	81	51	61.4%	2.729	(1.898-3.956)	1.2E-08	1.29E-07
Crevaviridae	38	344	9.9%	0.188	(0.13-0.263)	3.1E-33	3.45E-32
Inoviridae	1	53	1.9%	0.032	(0.001-0.188)	6.9E-10	7.58E-09
Winoviridae	129	85	60.3%	2.612	(1.97-3.479)	3.8E-12	4.18E-11
Other	11	44	20.0%	0.428	(0.199-0.843)	1.1E-02	1.22E-01
Higher order	5 671	8 380	40.4%	1.232	(1.183-1.282)	3.4E-24	3.75E-23
Unknown	12 152	20 753	36.9%	1.010	(0.972-1.05)	6.1E-01	1.00E+00

*Two-sided Fisher exact tests were performed for one taxonomical family (or the categories Other, Higher order or Unknown) versus all others combined. Both nominal and FDR-corrected p-values are reported

Supplementary Table 5: Baseline characteristics of study participants (n=1034)*

**Demography variables**			
Age category	50-60 years	n (%)	181 (17.5)
	60-70 years	n (%)	481 (46.5)
	70-80 years	n (%)	372 (36.0)
Sex	Male	n (%)	582 (56.3)
	Female	n (%)	452(43.7)
Region	Region 1 (Moss)	n (%)	568 (54.9)
	Region 2 (Bærum)	n (%)	466 (45.1)
Nationality	Native	n (%)	919 (90.5)
	Non-native	n (%)	70 (6.9)
	Missing	n (%)	26 (2.6)
Marital status	Married/cohabiting	n (%)	811 (79.9)
	Not married/non-cohabiting	n (%)	202 (19.9)
	Missing	n (%)	2 (0.2)
Education	Primary school	n (%)	196 (19.3)
	High school	n (%)	393 (38.7)
	University/college	n (%)	419 (41.3)
	Missing	n (%)	7 (0.7)
Working status	Employed	n (%)	322 (31.7)
	Retired/unemployed	n (%)	566 (55.8)
	Other	n (%)	127 (12.5)
**Lifestyle variables**			
Overall HLI	Points	median (p25, p75)	3.5 (2.8, 4.3)
Smoking status	Non-smoker	n (%)	744 (73.3)
	Smoker	n (%)	269 (26.5)
	Missing	n (%)	2 (0.2)
Snus status	Non-snuser	n (%)	90 (88.8)
	Snuser	n (%)	70 (6.9)
	Missing	n (%)	44 (4.3)
BMI	kg/m2	median (p25, p75)	26.5 (24.1, 29.3)
Physical activity	min/week	median (p25, p75)	105 (0, 300)
Antibiotic usage	No	n (%)	828 (81.6)
	Yes	n (%)	136 (13.4)
	Unknown	n (%)	51 (5.0)
Antacid usage	No	n (%)	701 (69.1)
	Yes	n (%)	269 (26.5)
	Unknown	n (%)	45 (4.4)
**Diet variables**			
Energy, kcal/day	kcal/day	median (p25, p75)	2170.4 (1737.3, 2666.1)
Protein	E%	median (p25, p75)	16.6 (15.1, 18.2)
Carbohydrates	E%	median (p25, p75)	
Added sugar	E%	median (p25, p75)	4.1 (2.6, 6.5)
Fibre, g/day	kcal/day	median (p25, p75)	27.6 (21.8, 35.1)
Fat	E%	median (p25, p75)	34.5 (31.1, 37.9)
SFA	E%	median (p25, p75)	11.8 (10.2, 13.5)
MUFA	E%	median (p25, p75)	12.8 (11.4, 14.6)
PUFA	E%	median (p25, p75)	6.2 (5.3, 7.3)
TFA	E%	median (p25, p75)	0.3 (0.2, 0.4)
Alcohol	g/day	median (p25, p75)	8.7 (2.4, 18.8)

Abbreviations: BMI; body mass index, E%; energy percentage, g; gram, MUFA; monounsaturated fatty acids, n; number, PUFA; polyunsaturated fatty acids, SFA; saturated fatty acids, TFA; trans-fatty acids

* Values are median (p25, p75) for continuous variables and n (%) for categorical variables. The numbers available for analysis vary by variable.

Supplementary Table 6. Host variable associations with gut virome beta diversity.

host variable	n	df	R2	parOmegaSq	p
Sex	1034	1	0.001976	0.001008	0.001
Age	1034	2	0.00244	0.0005044	0.003
Region	1034	1	0.001599	0.0006308	0.001
BMI	623	1	0.003451	0.001843	0.001
Physical activity	728	1	0.003303	0.001928	0.001
Smoking	1013	1	0.002776	0.001788	0.001
Snus	971	1	0.001209	0.000178	0.089
Education	1008	2	0.003136	0.001151	0.001
Marital status	1013	1	0.001484	0.0004958	0.003
Working status	1015	2	0.003608	0.001637	0.001
Nationality	989	1	0.002425	0.001413	0.001
Energy (kcal/day)	630	1	0.002492	0.0009021	0.003
Protein (E%)	630	1	0.001295	-0.0002943	0.961
Carbohydrates (E%)	630	1	0.002136	0.0005459	0.013
Added sugar (E%)	630	1	0.00177	0.0001799	0.159
Fiber (g/day)	634	1	0.003711	0.002131	0.001
Fat (E%)	630	1	0.001562	-0.00002798	0.515
SFA (E%)	630	1	0.001727	0.0001369	0.217
MUFA (E%)	630	1	0.001937	0.0003472	0.047
PUFA (E%)	630	1	0.001868	0.0002778	0.064
TFA (E%)	630	1	0.001591	0.0000008423	0.453
Alcohol (g/day)	634	1	0.002878	0.001297(9)	0.001
HLI	615	1	0.004264	0.002635	0.001
Antibiotics use	964	1	0.001383	0.0003445	0.018
Antacid use	970	1	0.001219	0.0001874	0.072

*PERMANOVA test, nominal p-values are reported.

Supplementary Table 7: Software, algorithms and databases

Tool	Version	url
Data processing		
Metagenome-Atlas	2.4.3	https://github.com/metagenome-atlas/atlas
VirSorter	2.2.2	https://github.com/jiarong/VirSorter2
CheckV	0.8.1	https://bitbucket.org/berkeleylab/checkv/src/master/
Galah	0.3.1	https://github.com/wwood/galah
BBMap	38.96	https://sourceforge.net/projects/bbmap/
SAM tools	1.15.1	https://sourceforge.net/projects/samtools/files/samtools/1.15.1/
vConTACT	0.11.0	https://bitbucket.org/MAVERICLab/vcontact2/src/master/
Prodigal	2.6.3	https://github.com/hyattpd/Prodigal
DRAMv	1.4.1	https://github.com/WrightonLabCSU/DRAM
graphanalyzer	1.4.6	https://github.com/lazzarigioele/graphanalyzer
Workflow management tools		
Snakemake		https://snakemake.github.io/
Visualization tools		
Cytoscape	3.9.0	https://cytoscape.org
Statistics		
R package *vegan*	2.6.2	https://github.com/vegandevs/vegan
R package *MaAsLin2*	1.12	https://github.com/biobakery/Maaslin2
R package *micEco*	0.9.15	https://github.com/Russel88/MicEco
Databases	Date	
Pfam	15.11.2021	https://www.ebi.ac.uk/interpro/
CAZy	08.09.2022	http://www.cazy.org/
VOGDB	11.05.2022	https://vogdb.org/
KOfam	31.10.2022	https://www.genome.jp/tools/kofamkoala/
CAN	08.09.2022	https://bcb.unl.edu/dbCAN/
RefSeq	15.09.2022	https://www.ncbi.nlm.nih.gov/refseq/
INPHARED	13.06.2023	https://github.com/RyanCook94/inphared
Virus-Host DB	11.03.2023	https://www.genome.jp/virushostdb/
Virsorter	15.11.2020	https://osf.io/v46sc/download
CheckV	06.02.2021	https://portal.nersc.gov/CheckV/

Supplementary Table 8: Viral genomes identified in standard community

vOTU id	Closer reference	Host	Class	Family	Genome length, bp	Provirus	Genes	Viral genes	Quality check	Completeness	Completeness method
vOTU_Zymo1	*Salmonella* phage *epsilon34*	*Salmonella*	*Caudoviricetes*	Unclassified	41 219	Yes	141	45	Complete	100	Provirus (high-confidence)
vOTU_Zymo2	*Listeria* phage *B054*	*Listeria*	*Caudoviricetes*	Unclassified	50 835	Yes	102	31	High-quality	100	AAI-based (high-confidence)
vOTU_Zymo3	*Pseudomonas* phage *AUS531phi*	*Pseudomonas*	*Caudoviricetes*	Unclassified	37 690	Yes	139	31	Medium-quality	69.24	AAI-based (high-confidence)
vOTU_Zymo4	*Salmonella* phage *SEN34*	*Salmonella*	*Caudoviricetes*	Unclassified	26 937	Yes	40	26	Medium-quality	63.86	AAI-based (high-confidence)
vOTU_Zymo5	*Escherichia* phage *vB_EcoS-673R7*	*Escherichia*	*Caudoviricetes*	Unclassified	26 951	Yes	59	21	Medium-quality	51.54	AAI-based (high-confidence)
vOTU_Zymo6	*Pseudomonas* phage *H71*	*Pseudomonas*	*Caudoviricetes*	Unclassified	38 214	Yes	132	37	High-quality	97.36	AAI-based (high-confidence)
vOTU_Zymo7	*Bacillus* phage *rho14*	*Bacillus*	*Caudoviricetes*	Unclassified	40 004	Yes	91	27	Complete	100	Provirus (high-confidence)
vOTU_Zymo8	*Enterococcus* phage *SEsuP-1*	*Enterococcus*	*Caudoviricetes*	Unclassified	31 702	Yes	101	24	Complete	100	Provirus (medium-confidence)
vOTU_Zymo9	*Escherichia* phage *phiSTEC1575-Stx2k*	*Escherichia*	*Caudoviricetes*	Unclassified	35 029	Yes	88	31	Medium-quality	71.9	AAI-based (high-confidence)
vOTU_Zymo10	*Escherichia* phage *CMS-2020a*	*Escherichia*	*Caudoviricetes*	Unclassified	55 040	No	76	25	Medium-quality	50.51	AAI-based (high-confidence)
vOTU_Zymo11	*Bacillus* phage *PBP180*	*Bacillus*	*Caudoviricetes*	Unclassified	33 322	Yes	56	22	Medium-quality	61.87	AAI-based (high-confidence)
vOTU_Zymo12	*Listeria* phage *HB17054*	*Listeria*	*Caudoviricetes*	Unclassified	39 728	Yes	135	33	High-quality	98.8	AAI-based (high-confidence)
vOTU_Zymo13	*Klebsiella* phage *KP12*	*Klebsiella*	n.a.	Unclassified	20 604	Yes	38	16	Medium-quality	55.56	AAI-based (medium-confidence)
vOTU_Zymo14	*Pseudomonas* phage *F10*	*Pseudomonas*	*Caudoviricetes*	Unclassified	40 977	Yes	112	34	Complete	100	Provirus (high-confidence)
vOTU_Zymo15	*Pseudomonas* phage *PP9W2*	*Pseudomonas*	*Caudoviricetes*	Unclassified	59 170	Yes	174	54	Complete	100	Provirus (high-confidence)
