# Supplementary material for: Exploring the gut DNA virome in fecal immunochemical test stool samples reveals associations with lifestyle in a large population-based study
Source: Nat Commun. 2024 Feb 29;15:1791. doi: 10.1038/s41467-024-46033-0 (PMC10904388; doi:10.1038/s41467-024-46033-0)
Supplement: Supplementary file 3 — Description of Additional Supplementary Files [file 41467_2024_46033_MOESM3_ESM.pdf]

File Name: Supplementary Data 1
Description: Characteristics of identified viral genomes

File Name: Supplementary Data 2
Description: Host variable associations with gut virome alpha diversity

File Name: Supplementary Data 3
Description: Host variable associations with gut viral abundance
