# Supplementary material for: Exploring the gut DNA virome in fecal immunochemical test stool samples reveals associations with lifestyle in a large population-based study
Source: Nat Commun. 2024 Feb 29;15:1791. doi: 10.1038/s41467-024-46033-0 (PMC10904388; doi:10.1038/s41467-024-46033-0)
Supplement: Supplementary file 7 — Reporting Summary [file 41467_2024_46033_MOESM7_ESM.pdf]

nature portfolio

Corresponding author(s): Trine B Rounge
Last updated by author(s): Jan 22, 2024

Reporting Summary

Nature Portfolio wishes to improve the reproducibility of the work that we publish. This form provides structure for consistency and transparency in reporting. For further information on Nature Portfolio policies, see our Editorial Policies and the Editorial Policy Checklist.

Statistics

For all statistical analyses, confirm that the following items are present in the figure legend, table legend, main text, or Methods section.

n/a	Confirmed	
☐	☒	The exact sample size (*n*) for each experimental group/condition, given as a discrete number and unit of measurement
☐	☒	A statement on whether measurements were taken from distinct samples or whether the same sample was measured repeatedly
☐	☒	The statistical test(s) used AND whether they are one- or two-sided<br>*Only common tests should be described solely by name; describe more complex techniques in the Methods section.*
☐	☒	A description of all covariates tested
☐	☒	A description of any assumptions or corrections, such as tests of normality and adjustment for multiple comparisons
☐	☒	A full description of the statistical parameters including central tendency (e.g. means) or other basic estimates (e.g. regression coefficient) AND variation (e.g. standard deviation) or associated estimates of uncertainty (e.g. confidence intervals)
☐	☒	For null hypothesis testing, the test statistic (e.g. *F*, *t*, *r*) with confidence intervals, effect sizes, degrees of freedom and *P* value noted<br>*Give P values as exact values whenever suitable.*
☒	☐	For Bayesian analysis, information on the choice of priors and Markov chain Monte Carlo settings
☒	☐	For hierarchical and complex designs, identification of the appropriate level for tests and full reporting of outcomes
☐	☒	Estimates of effect sizes (e.g. Cohen's *d*, Pearson's *r*), indicating how they were calculated

Our web collection on statistics for biologists contains articles on many of the points above.

Software and code

Policy information about availability of computer code

Data collection	no software was used
Data analysis	Metagenome-Atlas 2.4.3. VirSorter 2.2.2, CheckV 0.8.1, Galah 0.3.1, BBMap 38.96, SAM tools1.15.1, vConTACT 0.11.0, Prodigal 2.6.3, DRAMv 1.4.1, ,graphanalyzer 1.4.6, Snakemake, Cvtoscape v 3.9.0, R-vegan 2.6.2, R-MaAslin2 1.12, R-micEco 0.9.15, custom: VirMake (https://github.com/uio-bmi/VirMake), custom scripts (available at https://github.com/Rounge-lab/CRCbiome_virome_2023)<br><br>Versions of all tools and databases are provided in the Supplementary Table 7.

For manuscripts utilizing custom algorithms or software that are central to the research but not yet described in published literature, software must be made available to editors and reviewers. We strongly encourage code deposition in a community repository (e.g. GitHub). See the Nature Portfolio guidelines for submitting code & software for further information.

Data

Policy information about availability of data

All manuscripts must include a data availability statement. This statement should provide the following information, where applicable:

- Accession codes, unique identifiers, or web links for publicly available datasets
- A description of any restrictions on data availability
- For clinical datasets or third party data, please ensure that the statement adheres to our policy

DNA sequencing data generated in this study have been deposited in the database Federated EGA https://ega-archive.org/. Per participant consent, submitted FASTQ files exclude reads mapping to the human genome. The data are available under restricted access due to the sensitive nature of data derived from human subjects. Processing of data from this study must comply with the General Data Protection Regulation (GDPR). Access can be obtained by following the procedure described here: https://www.mn.uio.no/sbi/english/groups/rounge-group/crcbiome/. Requests for data access can also be directed to Trine B Rounge, trinro@uio.no.
The processed FASTA files of CRCbiome vOTUs detected in 5 or more individuals are available at the European Nucleotide Archive with accession number ERS16322857. The data on genome length, prevalence, taxonomy assignment of all vOTUs generated in this study, and data on their correlation to lifestyle and demographic factors, are provided in the Supplementary Data. A minimum dataset for reproduction of analyses is available at https://github.com/Rounge_lab/CRCbiome_virome_2023. Links to publicly available databases used in this study are provided in Supplementary Table 7.

Links to publically available databases used in the study - Pfam: https://www.ebi.ac.uk/interpro/; CAZy: http://www.cazy.org/; VOGDB: https://vogdb.org/; KOfam: https://www.genome.jp/tools/kofamkoala/; CAN: https://bcb.unl.edu/dbCAN/; RefSeq: https://www.ncbi.nlm.nih.gov/refseq/; INPHARED: https://github.com/RyanCook94/inphared/; Virus-Host DB: https://www.genome.jp/virushostdb/; Virsorter: https://osf.io/v46sc/download; CheckV: https://portal.nersc.gov/CheckV/
All the links are also provided in the Supplementary Table 10.

Research involving human participants, their data, or biological material

Policy information about studies with human participants or human data. See also policy information about sex, gender (identity/presentation), and sexual orientation and race, ethnicity and racism.

Reporting on sex and gender	The study comprised 582 males (56.3 %) and 452 females (43.7 %). The data on sex were retrieved from the national population registry. All participants signed an informed consent which included permission to retrieve information from national registries. No data on gender was collected or analyzed. Sex data were used as an outcome variable in the differential vOTU abundance and alpha- and beta-diversity analyses.
Reporting on race, ethnicity, or other socially relevant groupings	Self-reported information on nationality (native Norwegian/non-native heritage), marital status (married or cohabiting/not married and not cohabiting), level of education (primary school/high school/university or college) and working status (employed/retired or unemployed) was collected. These data were used as outcome variables in the differential abundance and alpha- and beta-diversity analyses.
Population characteristics	The CRCbiome enrolled individuals aged 55-76 who had a positive FIT test, were scheduled for the subsequent colonoscopy and had no previous medical record of colorectal cancer. After colonoscopy, participants were grouped into four main categories: no confirmed neoplastic findings (n = 169); non-advanced lesions (n = 423); advanced lesions (n = 376); and CRC (n = 66)
Recruitment	The recruitment protocol, as well as inclusion and exclusion criteria for the CRCbiome cohort, are provided in detail in the CRCbiome study design paper (https://bmccancer.biomedcentral.com/articles/10.1186/s12885-021-08640-8). To comply with the overall aim of the CRCbiome study, the proportion of individuals with premalignant or malignant colorectal cancer lesions was higher than in the general population. Sensitivity analyses excluding participants with a malignancy did not impact the outcomes reported in this paper.<br>Seven individuals , whose stool samples were used for the FIT stability testing, were recruited in the frame of the regular Piedmont Region CRC screening in the Microbiome and MiRNA in Torino Screening (MITOS) project (https://www.gastrojournal.org/article/S0016-5085(23)00811-9/fulltext). Within this screening program, all residents aged 59–69 are invited to undergo a single sample biennial FIT.
Ethics oversight	The CRCbiome project was approved by the Norwegian Regional Committees for Medical and Health Research Ethics (Approval no.: 63148). The MITOS cohort project was approved by the local Ethics committee (AOU Città della salute e della Scienza di Torino, Italy; Approval no.: 0061857).

Note that full information on the approval of the study protocol must also be provided in the manuscript.

Field-specific reporting

Please select the one below that is the best fit for your research. If you are not sure, read the appropriate sections before making your selection.

☒ Life sciences ☐ Behavioural & social sciences ☐ Ecological, evolutionary & environmental sciences

For a reference copy of the document with all sections, see nature.com/documents/nr-reporting-summary-flat.pdf

Life sciences study design

All studies must disclose on these points even when the disclosure is negative.

Sample size	Details on the sample size selection are provided in the CRCbiome study design paper (https://bmccancer.biomedcentral.com/articles/10.1186/s12885-021-08640-8). No other sample size considerations were made for this paper. The final dataset included 1034 individuals.
Data exclusions	Samples with <0.7 ng/ul DNA concentration (n = 14), or a depth of <1Gb sequencing data after quality filtering (n = 9) were excluded from the analysis.
Replication	The ZymoBIOMICS Microbial Community Standard was used as a positive control (n = 2). We reproducibly generated microbial profiles for these samples, including 15 viruses that target community bacteria. The information on detected viral genomes is now provided in Supplementary Table 8. Negative controls for DNA extraction (n = 6) and for library prep (n = 2) yielded a total of 32 and 3 QC sequencing reads respectively. All replicates were successfull.
Randomization	no randomization was performed for this descriptive study
Blinding	The lab personnel was blinded to group allocations during the DNA extraction, sequencing library prep and sequencing. The investigators were blinded to group allocations during the sequence data processing, vOTU generation and relative abundance estimation.

Reporting for specific materials, systems and methods

We require information from authors about some types of materials, experimental systems and methods used in many studies. Here, indicate whether each material, system or method listed is relevant to your study. If you are not sure if a list item applies to your research, read the appropriate section before selecting a response.

Materials & experimental systems

n/a	Involved in the study	
☒	☐	Antibodies
☒	☐	Eukaryotic cell lines
☒	☐	Palaeontology and archaeology
☒	☐	Animals and other organisms
☐	☒	Clinical data
☒	☐	Dual use research of concern
☒	☐	Plants

Methods

n/a	Involved in the study	
☒	☐	ChIP-seq
☒	☐	Flow cytometry
☒	☐	MRI-based neuroimaging

Clinical data

Policy information about clinical studies

All manuscripts should comply with the ICMJEguidelines for publication of clinical research and a completedCONSORT checklist must be included with all submissions.

Clinical trial registration	The BCSN study (parent study to the CRCbiome): NCT01538550
Study protocol	The CRCbiome (nested within BCSN study): https://bmccancer.biomedcentral.com/articles/10.1186/s12885-021-08640-8<br>The BCSN study: https://classic.clinicaltrials.gov/ct2/show/NCT01538550
Data collection	Locales: two hospital catchment areas in South-East Norway; recruitment: 2017-March 2021; data collection: 2017-2021
Outcomes	Primary outcome of the CRCbiome study relates to the colonoscopy result. For this paper, no outcome was defined.

Plants

Seed stocks	*Report on the source of all seed stocks or other plant material used. If applicable, state the seed stock centre and catalogue number. If plant specimens were collected from the field, describe the collection location, date and sampling procedures.*
Novel plant genotypes	*Describe the methods by which all novel plant genotypes were produced. This includes those generated by transgenic approaches, gene editing, chemical/radiation-based mutagenesis and hybridization. For transgenic lines, describe the transformation method, the number of independent lines analyzed and the generation upon which experiments were performed. For gene-edited lines, describe the editor used, the endogenous sequence targeted for editing, the targeting guide RNA sequence (if applicable) and how the editor was applied.*
Authentication	*Describe any authentication procedures for each seed stock used or novel genotype generated. Describe any experiments used to assess the effect of a mutation and, where applicable, how potential secondary effects (e.g. second site T-DNA insertions, mosiacism, off-target gene editing) were examined.*
